# Class I TCP in fruit development: much more than growth

**DOI:** 10.3389/fpls.2024.1411341

**Published:** 2024-05-28

**Authors:** Yushuo Gao, Farid Regad, Zhengguo Li, Julien Pirrello, Mondher Bouzayen, Benoît Van Der Rest

**Affiliations:** ^1^Laboratoire de Recherche en Sciences Veígeítales - Génomique et Biotechnologie des Fruits, Universiteí de Toulouse, Centre national de la recherche scientifique (CNRS), Université Toulouse III - Paul Sabatier (UPS), Toulouse-Institut National Polytechnique (INP), Toulouse, France; ^2^Key Laboratory of Plant Hormones and Development Regulation of Chongqing, School of Life Sciences, Chongqing University, Chongqing, China; ^3^Center of Plant Functional Genomics, Institute of Advanced Interdisciplinary Studies, Chongqing University, Chongqing, China

**Keywords:** teosinte branched, cycloidea, proliferating cell factor (TCP), class I TCP, transcription factor, fruit development, fruit ripening

## Abstract

Fruit development can be viewed as the succession of three main steps consisting of the fruit initiation, growth and ripening. These processes are orchestrated by different factors, notably the successful fertilization of flowers, the environmental conditions and the hormones whose action is coordinated by a large variety of transcription factors. Among the different transcription factor families, TEOSINTE BRANCHED 1, CYCLOIDEA, PROLIFERATING CELL FACTOR (TCP) family has received little attention in the frame of fruit biology despite its large effects on several developmental processes and its action as modulator of different hormonal pathways. In this respect, the comprehension of TCP functions in fruit development remains an incomplete puzzle that needs to be assembled. Building on the abundance of genomic and transcriptomic data, this review aims at collecting available *TCP* expression data to allow their integration in the light of the different functional genetic studies reported so far. This reveals that several Class I *TCP* genes, already known for their involvement in the cell proliferation and growth, display significant expression levels in developing fruit, although clear evidence supporting their functional significance in this process remains scarce. The extensive expression data compiled in our study provide convincing elements that shed light on the specific involvement of Class I *TCP* genes in fruit ripening, once these reproductive organs acquire their mature size. They also emphasize their putative role in the control of specific biological processes such as fruit metabolism and hormonal dialogue.

## 1 Introduction

In addition to their major importance in human nutrition, fruits have become a vast area of investigation in recent decades given their contribution to the understanding of the evolutionary history of angiosperms (Seymour et al., 2013) through their role in seed development and dispersal. A deeper understanding of the mechanisms behind the development and ripening of fleshy fruits is of major interest considering the wide range of applications it opens up for plant breeding, improvement of quality attributes and post-harvest handling as well as technological value. Transcription factors (TFs) are instrumental in mediating the transcriptomic reprogramming driving the developmental processes underpinning organ and tissue differentiation. Among the many TF families involved in fruit development, MADS-box TFs have been documented as the core contributors, as evidenced by the role of the master regulators, RIN, TAGL1, and MBP3 in the control of fruit ripening and locular gel differentiation in tomato (Vrebalov et al., 2002, 2009; Zhang et al., 2019b; Huang et al., 2021). The involvement of other TF families such as NAC, ERF, ARF or DOF has also been well documented (Hao et al., 2015; Liu et al., 2020; Forlani et al., 2021; Chirinos et al., 2023; Zou and Sun, 2023) and likewise, MYB TFs have been reported to actively contribute to fruit quality through the control of texture, flavor and phenolic and carotenoid metabolism (Allan and Espley, 2018). Overall, it is now well established that fleshy fruit development is made of a series of developmental transitions involving almost all types of TF families even though little is known about the specific role of many of these transcriptional regulators. In this view, the TCP transcription family exemplifies this lack of knowledge. Members of this transcription family genes have largely been described and reviewed for their effect on several developmental processes such as cell growth and proliferation, germination, embryogenesis, leaf and flower morphogenesis, biotic and abiotic stress responses (Dhaka et al., 2017; Lan and Qin, 2020; Viola and Gonzalez, 2023; Viola et al., 2023). In the previous decade, the active expression of *TCP* genes has been reported in a large number of species cultivated for its fruits such as tomato (Parapunova et al., 2014), *Prunus mume* (Zhou et al., 2016), strawberry (Wei et al., 2016), grapevine (Jiu et al., 2019; Leng et al., 2019), banana (Wang et al., 2022a), apple (Tabarelli et al., 2022), eggplant (Li et al., 2022), citrus (Liu et al., 2022a), passion fruit (Rizwan et al., 2022), cucumber (Ren et al., 2023), kiwifruit (Li et al., 2024) and pepper (Dong et al., 2024). Despite these numerous inventories, the impacts of *TCP* on fruit development remain enigmatic.

The TCP gene family was first described as a small group of plant-specific transcription factors sharing a conserved domain, named TCP after the three first characterized family members, TEOSINTE BRANCHED (TB) 1 from maize, CYCLOIDEA (CYC) from *Antirrhinum majus*, and PROLIFERATING CELL FACTORS (PCFs) from rice (Cubas et al., 1999). The TCP domain consists of a non-canonical basic-helix-loop-helix (bHLH) structure that allows DNA binding and protein-protein interactions (Cubas et al., 1999; Nicolas and Cubas, 2016). The protein crystal structure of rice TCP OsPCF6 revealed that the TCP domain functions as a dimer, with two TCP domains closely intertwined into a stable conformation (Sun et al., 2020). In addition, the DNA-TCP-domain complex structures were determined on AtTCP15 and AtTCP10: the homodimeric TCP domains adopt a three-site recognition mode for double-stranded DNA through a short pair of β-strands formed in the dimer interface completed with two basic flexible loops from each monomer (Zhang et al., 2023). Based on phylogenetic analysis, TCPs can be classified into two homology classes: Class I and Class II with the latter being further divided into two subclasses: the CIN-TCP subclass and the CYC/TB1 subclass (Cubas et al., 1999). Members of Class I TCP display a shorter TCP domain with a 4 amino acid deletion in the basic region and also exhibit a conserved region close to the TCP domain C-terminal extremity (Zhou et al., 2022). Both CIN and CYC/TB1 proteins are highly conserved and have been shown to be involved in controlling leaf morphology and size, petal development, trichome formation, and plant flowering (Bresso et al., 2018; Sarvepalli and Nath, 2018; Lan and Qin, 2020; Rath et al., 2022). In addition, the expression of *CIN* genes is controlled by the well-conserved microRNA319 (Bresso et al., 2018). To our knowledge, few studies reported the involvement of Class II TCPs in fruit development or ripening; only Class II AtTCP4 was recently found to contribute to the determination of ovule identity (Lan et al., 2023) and to the specification of apical gynoecium (Wang et al., 2024). Therefore, the current review will focus on Class I TCP functions in the frame of fruit development and ripening following flower fertilization. It aims at integrating recent literature and available expression data on Class I TCPs in order to draw a first picture of the potential role of this class of transcription factors in fruit biology.

### 1.1 Expression patterns of Class I *TCP* genes suggesting multiple actions in fruit development and ripening

Over the past decade, NGS technologies have enabled the generation of ever-increasing amounts of genomic and transcriptomic data from a large panel of plant species. Taking advantage of these resources, we performed a comprehensive survey to gain insight into the expression patterns in fruit organs of TCP-encoding genes. Interestingly, monitoring the expression of Class I *TCP* TF genes either by qRT-PCR or by RNAseq in plant species representative of a large diversity of botanical families (**Table 1**), revealed that TCPs are expressed in several fleshy fruits such as the dicots apple, pear, strawberry, blueberry, cucumber, tomato and grape and the monocots banana.

**Table 1:** List of Class I TCP genes expression characterized in fruits.

Plant species	Class I TCP genes expression situation	References
Apple (*Malus domestica*)	1. Expressed in fruits and downregulated before ripening: *MdTCP22*, *MdTCP32* and *MdTCP47* 2. Expressed in fruits and upregulated before ripening: *MdTCP1*, *MdTCP13* and *MdTCP14*	(Xu et al., 2014)
Chinese White Pear (*Pyrus bretschneideri*)	1. Highly expressed in ovary: *PbTCP32* and *PbTCP33* 2. Highly expressed in fruits: *PbTCP2/5/7/12/13/17/21/23/24/34* (1) *PbTCP23* and *PbTCP24* increased firstly and then decreased during fruit development (2) *PbTCP7* reached a peak at 63 DAP	(Zhao et al., 2021)
Strawberry (*Fragaria vesca*)	1. Preferentially expressed in half red fruits: *FvTCP19* 2. Preferentially expressed in ripe fruits: *FvTCP9, FvTCP12* and *FvTCP17*	(Wei et al., 2016)
Blueberry (*Vaccinium* spp.)	1. Preferentially expressed in green fruits: *VcTCP31*, *VcTCP2*, *VcTCP8*, *VcTCP43*, and *VcTCP38* 2. Preferentially expressed in ripening fruits (pink fruits): *VcTCP60*, *VcTCP4*, and *VcTCP54*	(Li et al., 2021)
Tomato (*Solanum lycopersicum*)	1. Expressed in the whole berry development process: *SlTCP27* 2. Preferentially expressed during fruit ripening: *SlTCP12, SlTCP15, SlTCP18*	(Parapunova et al., 2014)
Grape (1) (*V. vinifera and Vitis labrusca* *cv. “Jumeigui”*)	1. Preferentially expressed in growing green berries: *VvTCP7, 9, 14 and 16* 2. Preferentially expressed in immature green berries: *VvTCP3, 6, 14, 15* 3. Preferentially expressed in ripe berries: *VvTCP17* 4. Expressed in the whole berry development process: *VvTCP5*	(Jiu et al., 2019)
Grape (2) (*Vitis vinifera* *cv. “Corvina”*)	1. Preferentially expressed in green fruits: *VvTCP2, 3, 4, 7, 9, 12* and *16* 2. Preferentially expressed at veraison or in ripe fruits: *VvTCP17* and *VvTCP13*	(Leng et al., 2019)
Cucumber (*Cucumis sativus* L.)	1. Preferentially expressed in carpel: *CsTCP1*, *CsTCP15*, *CsTCP16*, *CsTCP18* and *CsTCP19*	(Wen et al., 2020)
Banana *(Musa acuminata* L.*)*	1. Expressed during fruit development (0 and 20 DAF): *MaPCF1, MaPCF2, MaPCF4, MaPCF7, MaPCF10, MaPCF11, MaPCF12, MaPCF13, MaPCF19, MaPCF20, MaPCF22, MaPCF24, MaPCF25* and *MaPCF27* 2. Expressed during fruit ripening (80 DAF): *MaPCF2, MaPCF4, MaPCF11, MaPCF18, MaTCP24* and *MaPCF26* 3. Expressed during post-harvest stages (8 and 14 DPH): *MaPCF2, MaPCF8, MaPCF10* and *MaPCF26*	(Wang et al., 2022a)

**Figure 1:**
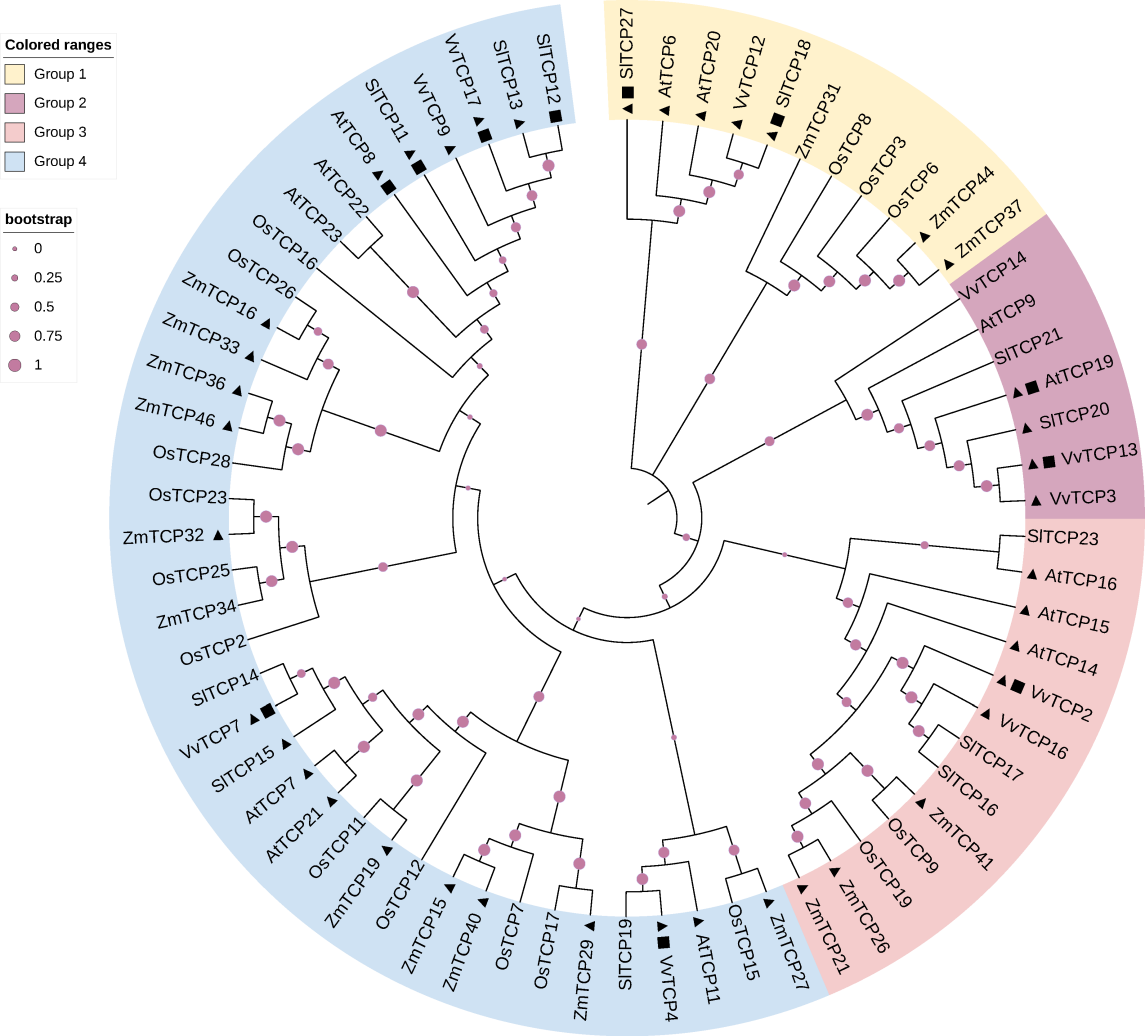
Phylogenetic tree of Class I TCP proteins. The phylogenetic analysis of *TCP* gene family among grape (*Vitis vinifera)*, Arabidopsis (*Arabidopsis thaliana)*, rice (*Oryza sativa)*, maize (*Zea mays*), and tomato (*Solanum lycopersicum*). The circular phylogenetic tree was constructed using the full-length protein sequences of TCPs from five species by MEGAX using the MUSCLE alignment and Neighbor-Joining (NJ) method. Bootstrap analysis was performed using 1000 replicates. Triangles represent genes specifically expressed in the early stages of fruit development, and squares represent genes specifically expressed in the late stages of fruit development. Gene information shown in the **Supplementary Table S1**.

**Table 1** shows that several Class I *TCP* genes are preferentially expressed at early stages of fruit development while others exhibit higher expression levels at late developmental stages, from ripening initiation to full-ripe fruit (**Table 1**). To further refine the classification of the *TCP* genes inventoried in **Table 1**, we built a dedicated phylogenetic tree based on the genome annotations of five species representative of Angiosperm families: Arabidopsis, tomato, grapes, rice and maize (*Arabidopsis thaliana*, *Solanum lycopersicum, Vitis vinifera*, *Oryza sativa* and *Zea mays* respectively). The tree divides Class I *TCP* family into 4 sub-groups encompassing the majority of the annotated Class I *TCP* (**Figure 1**). Interestingly, these sub-groups can be associated to the presence or absence of conserved motifs predicted with the MEME software (Bailey and Elkan, 1994), thus consolidating the proposed phylogeny (**Supplementary Table S1**). When available, all the *TCP* genes listed in **Table 1** were classified using the groups deduced from the phylogeny (**Supplementary Table S2**). *TCP* genes from each group are found to be expressed during fruit development or ripening, thus suggesting a coordinated action of TCP factors as reported in other physiological processes in vegetative organs (Danisman, 2016; Viola et al., 2023).

**Figure 2:**
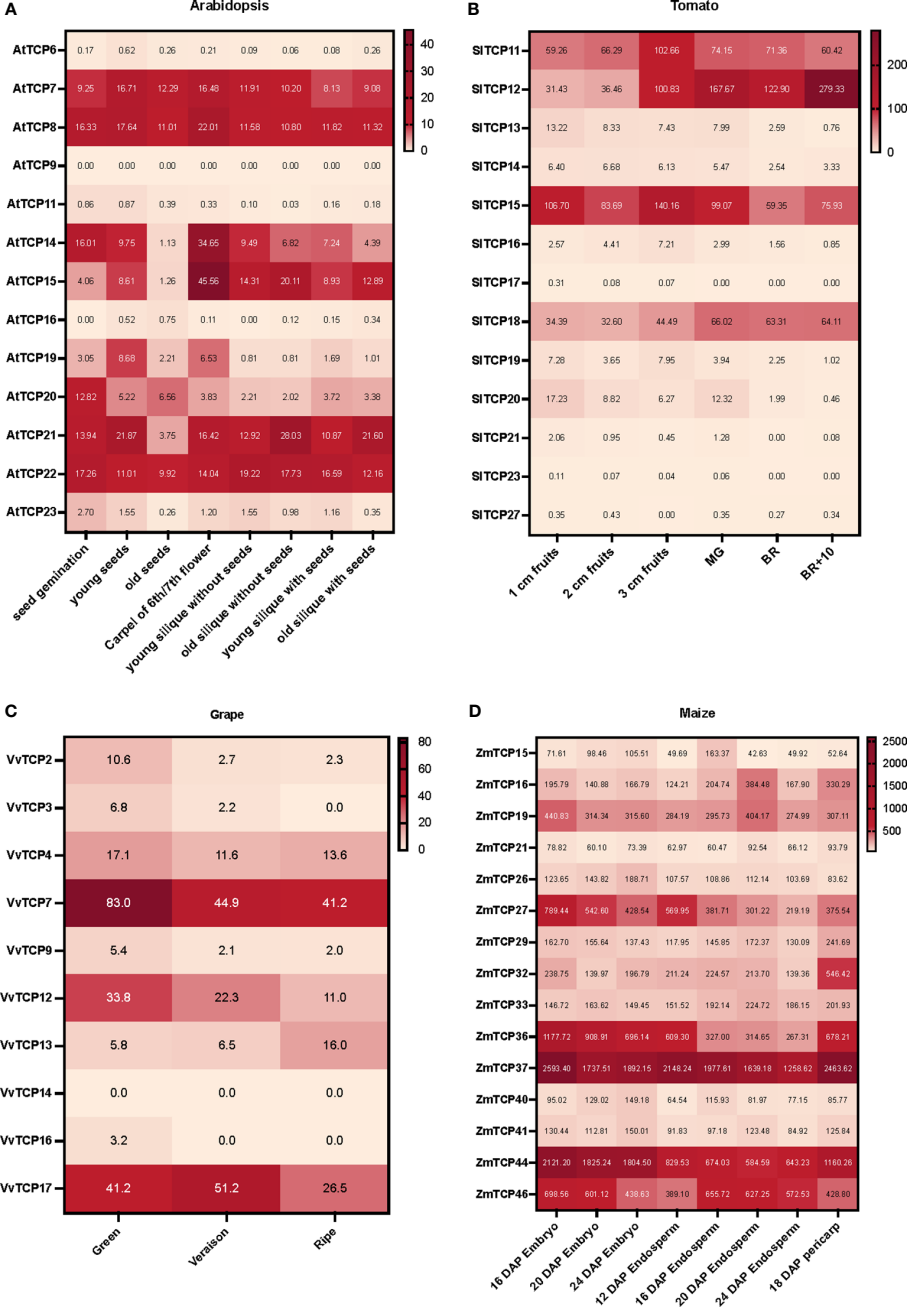
Heatmaps of transcript accumulation profiles of Class I *TCP* genes in **(A)** Arabidopsis (*Arabidopsis thaliana)*, **(B)** tomato (*Solanum lycopersicum*), **(C)** grape (*Vitis vinifera cv ‘Corvina’)*, and **(D)** maize (*Zea mays*). The transcript accumulation data were obtained from BAR-ePlant for maize and Arabidopsis, from TomExpress for tomato and from GEO DataSets for grapes. It was visualized as heat maps using Prism 9. The color scale represents the transcript levels with increased (red) or decreased (white) transcript abundance.

Massive amounts of transcriptomics data generated either by RNAseq or microarrays, can be easily accessed via different datamining and visualization platforms such as BAR-ePlant (The Bio-Analytic Resource for Plant Biology) (Waese et al., 2017), GEO DataSets (Barrett et al., 2009, 2011), TomExpress (Zouine et al., 2017) or Tomato Expression Atlas (TEA) (Fernandez-Pozo et al., 2017; Shinozaki et al., 2018). Notably, these platforms allow the normalization of the signals from large datasets, enabling the comparative assessment of expression levels in different conditions (including different development stages). We collected the expression patterns of Class I *TCP* genes from four plant species: tomato (*Solanum lycopersicum*) and grapes (*Vitis vinifera*) fleshy berries, maize (*Zea mays*) kernel and *Arabidopsis thaliana* dry silique. The expression data were extracted from BAR-ePlant for maize and Arabidopsis, from TomExpress for tomato and from GEO DataSets for grape and used to build heatmaps on representative stages for each fruit or imbedded seeds (**Figure 2**). For tomato, the interpretation of these heatmaps was completed by the analysis of TEA and TomExpress data to highlight the tissues and fruit developmental stages exhibiting preferential expression (**Supplementary Table S1**). These expression data suggest the involvement of several Class I *TCP* genes in fruit development, either at early (growth, immature fruit) or late stages (ripening, from its initiation to senescence). Among the *TCP* isoforms highly expressed in fruits, some display high expression levels in both vegetative and reproductive tissues (stems, shoot, leaves, seeds and fruits) while others exhibit preferential expression during fruit development or ripening (**Supplementary Table S1**). Notably, the *TCPs* expressed at the early stages of fruit development are also expressed in vegetative tissues. For instance, the Arabidopsis *AtTCP14* and *AtTCP15* are highly expressed in the carpel and the silique, but also in germinating seeds, shoot apex and leaves (**Figure 2A**; **Supplementary Table S1**). The expression of *SlTCP12, SlTCP15, SlTCP18* have been reported in tomato fruit ripening, with *SlTCP12* and *SlTCP18* expression being related to the actions of the master regulators of ripening *RIN, Cnr* and *AP2a* (Parapunova et al., 2014). Supporting these data, *SlTCP12* and *SlTCP18* display a preferential expression during fruit ripening (**Figure 2B**). By contrast, *SlTCP11* and *SlTCP15* genes are expressed in a wide variety of stages with a maximum in young fruits (3cm fruits in **Figure 2B**). Noteworthy, the *TCP* genes highly expressed in early fruit development exhibit a high expression in root, leaf, meristems and flower buds. More recently it was reported that SlTCP15 is a direct target of the kinase CDK8 and that the phosphorylated form is active in inducing pollen development (Xu et al., 2024). Altogether, these data suggest that SlTCP15 is involved in several developmental processes. In grape, Class I *TCP* genes are highly expressed in green berry (*VvTCP7* and *VvTCP12*) and exhibit also high expression in leaves and vegetative buds, while *VvTCP13* expression peaks only in ripe berries (**Figure 2C**). As for maize, the structure of the kernel with the fused pericarp and seed tegument did not allow to discriminate between early and late fruit development, although it appears that all the Class I *TCPs* expressed in endosperm growth display also expression in the pericarp (**Figure 2D**).

Highlighting the isoforms highly expressed in fruit development on the phylogenetic tree (**Figure 1**) failed to reveal any obvious correlation between the territories of expression and the clustering of the TCP isoforms within the different groups. Likewise, no obvious correlation was found when considering the expression studies reported in the literature (**Supplementary Table S2**). It is important to mention that the majority of Class I subclades are found in a wide range of land plant species, including the bryophyte *Sphagnum fallax* (Zhou et al., 2022), suggesting that unlike MADS-box transcription factors, the expression territory in reproductive organs is not a main driver of primary sequence evolution within Class I TCPs.

## 2 What is known about the functions of Class I TCP in fruit development?

The expression data presented in the first section enable to discriminate between the *TCPs* expressed in early fruit development encompassing the pre-mature stages and those expressed during the ripening phase. Remarkably, *TCP* genes expressed at early stages of fruit development display high expression level in vegetative tissues. In contrast, *TCPs* expressed at later stages of fruit development show greater tissue specificity and reach their expression peak during the ripening process.

Class I TCPs have been reported to act as modulators of cell proliferation, expansion and endoreplication, as component of hormone’s signaling cascades, often in response to environmental conditions, and also as regulators of leaf senescence or pigments accumulation (Viola et al., 2023). Very often, a single isoform can be involved in multiple mechanisms. For instance, *AtTCP15* was shown to operate in seed germination (Resentini et al., 2015), in auxin-mediated cell elongation (Ferrero et al., 2021), in cotyledon opening (Alem et al., 2022), in stamen development (Gastaldi et al., 2023), in the regulation of potato tuber sprouting via the modulation of the abscisic acid/gibberelic acid balance or in the modulation of anthocyanin biosynthesis (Viola et al., 2016; Ding et al., 2022; Wang et al., 2022b). Considering that all these mechanisms are also operating during fleshy fruit growth, tissues’ differentiation and ripening, Class I *TCPs* can be viewed as potential regulators of different aspects of fruit development.

### 2.1 Class I TCP factors in early fruit development

Early fruit development can be seen as a series of processes starting with the flower fertilization and leading to an immature fruit. These sequential developmental processes encompass the fruit set, fruit growth, tissue differentiation and modification of cell metabolism and coincides with seed formation and maturation. In fleshy fruits, the growth and accumulation of metabolites often occur in carpels, but can also be in inner tissues such as locular tissue, placenta and columella in tomato, or in seed outgrowth (like the *Litchi chinensis* aril) or in flower receptacle (like strawberry). In tomato, early fruit development includes three physiological processes: fruit set, cell division and cell expansion (Gillaspy et al., 1993). Interestingly, the fruit growth relies on a combination of cell divisions and cell expansion, the latter being boosted by endoreplication leading to an increase of cell ploidy mainly in pericarp and locular tissue cells (Azzi et al., 2015; Tourdot et al., 2023).

Given that Class I TCPs act as modulators of cell division and elongation in a wide range of biological processes, it is tempting to speculate on the potential conservation of this regulatory roles for *TCP* expressed during fruit development. In Arabidopsis, *AtTCP14*, *AtTCP15* and *AtTCP8* are known to activate several cell-cycle and cell-proliferation genes while inhibiting endoreplication, since modifications of ploidy number has been reported in various mutant backgrounds (Kieffer et al., 2011; Li et al., 2012; Peng et al., 2015; Zhang et al., 2019a). Interestingly, these three genes correspond to three Class I *TCP* genes highly expressed in the carpel and the silique (**Figure 2**). Although altered silique architecture has been reported in *AtTCP15* SRDX dominant negative lines, such as replum enlargement in plants (Uberti-Manassero et al., 2012), clear data demonstrating the impact of *AtTCPs* regulation on Arabidopsis silique development are still missing.

Evidence documenting the function of Class I *TCPs* in early fruit development is also limited in other species. In wheat (*Triticum aestivum* L.), the phenotype displayed by the *Ta-TCP9-A* mutants suggests the existence of an action of this gene on seed and fruit growth (Zhao et al., 2018). Indeed, two independent loss-of-function mutants carrying distinct mutations in *TaTCP9-A* gene display an increased grain length, width, and weight associated with an increase in the spike length. It is noteworthy that *Ta-TCP9* belongs to the same group than *AtTCP14* and *AtTCP15* (*Ta-TCP9* being close to *OsTCP9*, **Figure 1**), but its contribution to the regulation of the elongation processes occurring in the grain and the floral pieces still requires solid validation given that the mutant lines harbor several mutations in their genome in addition to the one affecting the *TCP9* gene.

*TCPs* have also received attention regarding their role in cotton fiber development from the ovule surface. *GhTCP14*, a Class I TCP also belonging to the *AtTCP14/AtTCP15* clade, was proposed to regulate fiber development, particularly during the initiation and elongation stages which coincides with early fruit ripening (Wang et al., 2013). *GhTCP14* action seems to interfere with auxin signaling and may involve other Class I *TCP* genes co-expressed with *GhTCP14*, including *GhTCP7a, 9b, 15a/b/c, 21*, and *22* (Li et al., 2017). This action can also be paralleled with the presumed function of *AtTCP14* and *AtTCP15* in trichomes’ formation (Camoirano et al., 2020).

### 2.2 Class I TCP factors in fruit ripening

Mining the transcriptomic data highlighted the expression of different *TCP* genes at different stages of fruit ripening (**Figure 2**), showing that some isoforms are preferentially expressed during the ripening process of tomato and grape fruit (see section 1). While similarities between fruit ripening and leaf senescence mechanisms have been suggested by several authors (Picton et al., 2011; Gapper et al., 2013; Wang et al., 2017; Ma et al., 2018, 2019), it would, however, be appropriate to distinguish between the ripening process sensu stricto and the over-ripening phase (Gapper et al., 2013). Obviously, both ripening and senescence involve large gene expression and metabolism reprogramming. A striking similarity between the two processes is the important role of the plant hormone ethylene, known to be a key regulator of the two processes (Picton et al., 2011; Sharma et al., 2021). Moreover, ABA, known as a plant senescence hormone, has been documented to impact fruit ripening (Ye et al., 2017; Kou et al., 2021; Gupta et al., 2022). At the cellular level, ripening and senescence share several common processes like chlorophyll breakdown and cell wall modifications (Forlani et al., 2019; Ling et al., 2021). In addition, several senescence-associated proteins are also associated with fruit ripening, including type 2C phosphatase SlPP2C (Jiang et al., 2023), isopentenyl transferase SlIPT4 (Zhang et al., 2018) and the recently characterized VviNAC60 in grape (D'Inca et al., 2023).

In the last decade, several studies have shed light on the regulatory effect exerted by TCP on leaf senescence, indicating that different TCP isoforms can act either as negative or positive regulators. For instance, overexpression of chrysanthemum gene *CmTCP14* in Arabidopsis results in delayed leaf senescence (Zhang et al., 2017), and AtTCP20 inhibits the expression of the lipoxygenase *AtLOX2*, encoding a key enzyme in jasmonic acid biosynthesis, thus leading to impaired leaf senescence (Danisman et al., 2012). Similarly, cabbage BrTCP21 delays leaf senescence by influencing the GA biosynthetic pathway (Xiao et al., 2019). By contrast, *BrTCP7* binds to the promoter regions of *BrOPR3* and *BrRCCR*, involved in jasmonic acid (JA) biosynthesis and chlorophyll breakdown respectively, thus suggesting that this TCP acts as a positive regulator of leaf senescence (Xu et al., 2019).

The study of *TCP* expression in fruit peach (*Prunus persicum*) development and ripening (Guo et al., 2018) highlighted the potential contribution of *PpTCP.A2*. Indeed, *PpTCP.A2* is expressed in fruit pre-ripening and its VIGS-induced silencing in peach fruit reveals an increase in ethylene production coinciding with an upregulation of *PpACS1*, encoding the enzyme that catalyzes the synthesis of ACC, the direct precursor of ethylene. The study suggested that the action of *PpTCP.A2* is reminiscent of *AtTCP20*, *CmTCP14* or *BrTCP21* action as negative regulator of leaf senescence (see above).

In banana (*Musa acuminata*), several *TCP* factors (*MaTCP15*, *MaTCP19*, *MaTCP20*, and *MaTCP22*) were found to be ethylene-inducible (Song et al., 2018). Interestingly, these authors suggested that *MaTCP20* could activate the promoters of two xyloglucan endotransglucosylase/hydrolases (*MaXTH10* and *MaXTH11*) since they contain the TCP-conserved (T/C) GGNCCCA regulatory elements and they can effectively bind *Ma*TCP20 protein as shown by EMSA and dual-luciferase assays. The data also suggest that *TCP* may impact fruit quality, since XTHs are involved in cell wall modification processes that accompany fruit softening.

Fruit color, an important quality trait, is controlled by *TCP* as suggested by different studies on anthocyanin accumulation. The overexpression of *FaTCP11* in strawberries (*Fragaria* × *ananassa*), a gene first identified by expression correlation network analysis on the transcriptomes of red ripe fruits from two parental lines and their progeny (Pillet et al., 2015), leads to enhanced expression of Leucoanthocyanidin Reductase (*LAR*) and Flavonone 3’-Hydroxylase (*F3’H*). This finding suggests that *FaTCP11* is critical in regulating the flavan-3-ols accumulation, thereby exerting a significant impact on strawberry quality. More recently the woodland strawberry *FvTCP9* gene was also shown to regulate positively anthocyanin accumulation using VIGS-induced RNAi silencing or gene overexpression (Xie et al., 2020). *FvTCP9* expression level is correlated with anthocyanin production, with the expression of phenolic-metabolism related genes and with that of other ripening-related genes involved in fruit softening or sugar accumulation.

Further sustaining the involvement of TCPs in the regulation of pigment accumulation in fleshy fruit species, the *MdTCP46* gene in apple (*Malus domestica*), has been reported to promote anthocyanin biosynthesis in response to high light exposure (An et al., 2020; You et al., 2023). Accordingly, overexpression of *MdTCP46* in fruit peel via infiltration effectively leads to a dramatic increase of anthocyanin production in fruit under high light. The same study demonstrated that *Md*TCP46 interacts with *Md*MYB1 and *Md*BT2 to form a regulatory module controlling anthocyanin accumulation under different light intensities. Interestingly, this regulatory module is also connected to hormone signaling cascades since MdTCP46 was shown to interact with the ABA signaling effectors *MdABI5* or MdRGL2a DELLA protein while conversely, its expression is inhibited by GA treatment (Liu et al., 2022b; You et al., 2023).

### 2.3 TCP regulation at the crossroad of hormonal signaling

The complex process of fruit development, from the fertilized flower to the final ripening phase, is regulated by an intricate interplay between multiple plant hormones. At early stages of fruit growth, gibberellic acid (GA) and auxin have synergistic action in promoting cell division and expansion, thereby regulating fruit development and enlargement after fertilization (Serrani et al., 2008; He and Yamamuro, 2022). Later, the transition from immature stage to ripening stage is characterized by shifts in phytohormone profiles such as auxin, ethylene and abscisic acid (ABA) accompanied by transcriptional reprogramming (Forlani et al., 2019). Several studies suggest that the hormonal regulation of the expression of *TCP* genes is a frequent and widespread phenomenon in plants despite the fact that the physiological significance of these TCPs remains obscure to date.

In tomato, *SlTCP12*, *SlTCP15* and *SlTCP18* genes that are expressed in fruit ripening display *Cis*-elements in their promoter regions shown to be typical binding sites of the RIN master regulator of fruit ripening as evidenced by yeast one-hybrid experiments (Parapunova et al., 2014).There are also reports supporting that ethylene acts as a direct or indirect regulator of *TCPs* expressed during fruit ripening. Indeed, co-expression studies demonstrated recently the existence of a strong correlation between the expression of *SlTCP15* and the ethylene-responsive transcription factor 4 *SlERF4* while a second correlation was observed between *SlTCP18* and Auxin-responsive Factor 5 (*SlARF5*), suggesting an interaction with multiple hormones signaling effectors (Edris et al., 2023).

In grape and blueberry, the expression patterns of *TCP* genes in fruits treated with different hormones also suggest a strong link between Class I *TCP* genes and hormone signaling. For instance, ABA treatment of grape berry at the veraison stage enhances *VvTCP14* and *VvTCP16* transcript levels while reducing that of *VvTCPs 6/7/15/17* (Jiu et al., 2019). On the other hand, in blueberry, expression of *VcTCP19/38/43/49/61* is sensitive to methyl-jasmonate treatment (Li et al., 2021).

## 3 Concluding remarks

The TCP family received less attention than many other transcriptions factor families like MADS, MYB, AP2-ERF, NAC with regard to their role in fruit biology. Yet, the increasing number of studies on different fruit species and the massive release of transcriptomic data produced by NGS technologies provided clues pointing to the putative involvement of Class I TCP transcription factors as active regulators of fruit development and ripening. While the puzzle is far from complete, the expression data reviewed in this manuscript support the idea that *TCP* genes expressed at early stages of fruit development, including growth and differentiation, correspond to those already shown to be involved in several growth or differentiation processes in vegetative organs. The exact nature of the processes involving TCP in fruit growth remains to be elucidated. Nevertheless, based on the current model related to germination or internode elongation, it is tempting to speculate on a potential role of TCPs from different phylogenetic groups on cell division, expansion or endoreplication. Among the *TCP* genes expressed during fleshy fruit development several display preferential expression at the ripening phase. While these genes still await functional characterization, studies in strawberry and banana suggest that TCPs can impact various determinants of fruit quality such as pigment accumulation or modification of texture associated with ripening. In addition, various studies suggest a link between TCPs and hormone signaling, notably ethylene, ABA and auxin, three phytohormones known as key regulators in the frame of fruit biology. Addressing the functional characterization of *TCP* genes in fruit will be a key challenge in the future, as it may open new avenues for improving fruit quality, and provide novel targets for breeding strategies or biotechnical engineering.

## Author contributions

YG: Conceptualization, Writing – original draft, Writing – review & editing. FR: Conceptualization, Formal Analysis, Writing – review & editing. LZ: Conceptualization, Formal Analysis, Validation, Writing – review & editing. JP: Formal Analysis, Validation, Writing – review & editing. MB: Conceptualization, Formal Analysis, Validation, Writing – review & editing, Writing – original draft. BV: Conceptualization, Validation, Writing – original draft, Writing – review & editing.

## Supplementary material

Supplementary Table 1 Inventory, classification and preferential expression of Class I TCP genes in Arabidopsis, tomato, grape and maize. Class I TCP proteins were classified into four groups based on protein sequences from *Arabidopsis thaliana, Solanum lycopersicum, Vitis vinifera*, and *Zea mays*. Expression data for each protein group were extracted from the following databases: BAR-ePlant, TEA, and TomExpress for *Solanum lycopersicum*; BAR-ePlant for *Arabidopsis thaliana* and *Zea may*; GEO DataSets for *Vitis vinifera*. Protein domains were predicted using the MEME software.

Supplementary Table 2 Classification of TCP isoforms described in the literature (listed in ). The categorization was deduced from a phylogenetic analysis performed on the MEGAX software on amino acids sequence using the MUSCLE alignment and the Neighbor Joining algorithm.
